# Ultrafast Miniature Robotic Swimmers with Upstream Motility

**DOI:** 10.34133/cbsystems.0015

**Published:** 2023-03-15

**Authors:** Yibin Wang, Hui Chen, Junhui Law, Xingzhou Du, Jiangfan Yu

**Affiliations:** ^1^ School of Science and Engineering, The Chinese University of Hong Kong, Shenzhen, China.; ^2^ Shenzhen Institute of Artificial Intelligence and Robotics for Society, Shenzhen, China.; ^3^ Department of Mechanical and Industrial Engineering, University of Toronto, Toronto, Canada.

## Abstract

With the development of materials science and micro–nano fabrication techniques, miniature soft robots at millimeter or submillimeter size can be manufactured and actuated remotely. The small-scaled robots have the unique capability to access hard-to-reach regions in the human body in a noninvasive manner. To date, it is still challenging for miniature robots to accurately move in the diverse and dynamic environments in the human body (e.g., in blood flow). To effectively locomote in the vascular system, miniature swimmers with upstream swimming capability are required. Herein, we design and fabricate a miniature robotic swimmer capable of performing ultrafast swimming in a fluidic environment. The maximum velocity of the swimmer in water is 30 cm/s, which is 60 body lengths. Moreover, in a tubular environment, the swimmer can still obtain a swimming velocity of 17 cm/s. The swimmer can also perform upstream swimming in a tubular environment with a velocity of 5 cm/s when the flow speed is 10 cm/s. The ultrasound-guided navigation of the swimmer in a phantom mimicking a blood vessel is also realized. This work gives insight into the design of agile undulatory milliswimmers for future biomedical applications.

## Introduction

Untethered miniature robots that can noninvasively access the enclosed space in the human body have been intensively investigated for their potential biomedical applications, such as minimally invasive surgery, thrombolysis, hyperthermia, and targeted drug delivery [1–5]. Different actuation strategies, including catalytic fuel [6], light [7], thermal [8], acoustic wave [9], electric field [10], and magnetic field [11–13], have been developed for the remote control of miniature robots. Among these proposed strategies, the magnetic field holds great promise in biomedical applications for it can harmlessly penetrate the human body. As a result, magnetic microrobots driven by magnetic torque and magnetic force have been developed. Based on their physical property, magnetic microrobots can be divided into synthetic rigid microrobots (e.g., magnetic roller [14] and magnetic helical microrobot [15,16]), microrobotic collectives (e.g., swarms of magnetic particles [17–21] and swarms of magnetic helical microswimmers [22]), biohybrid microrobots (e.g., magnetotactic bacteria [23]), and miniature soft robot (e.g., soft gripper [24] and soft magnetoelastic sheet [25,26]). Since the miniature soft robot is composed of materials with moduli close to those of soft biological tissues, they have the potential to facilitate the clinical translation of miniature robots for their enhanced biocompatibility [27–29].

Recently, one of the most marked challenges for medical robots is the precise navigation of the robot in complex physiological environments, especially in biofluids. Several miniature robots are designed to be implemented in biofluids. Yu et al. [18] explored the locomotion of the microrobotics swarm in biofluid and the bovine eyeball. Law et al. [30] achieved selective embolization in the blood vessel with the microrobotic swarm by applying the specially designed magnetic field. Yan et al. [31] developed a biohybrid magnetic helical microswimmer for in vivo image-guided therapy in gastric juice. Furthermore, some researchers have designed miniature robots capable of upstream swimming in fluidic flow. Alapan et al. [14] developed microrollers for targeted cargo delivery in blood flow, Ahmed et al. [32] developed an acousto-magnetic microswarm with upstream motility, Wang et al. [33] designed a soft undulatory swimmer that can resist flow in the intermediate flow regime, and Yang et al. [34] designed the unique spiral-rolling motion for the soft swimmer to swim against the flow. Yet, it is challenging to construct miniature robots that can effectively locomote against the flow with a flow speed similar to the physiological environment (up to 10 cm/s). To construct miniature soft robots with upstream motility, advanced structure design and actuation strategy need to be further investigated.

Herein, we design and fabricate a soft-bodied fish-like swimmer with nonuniform bending stiffness and streamlined body shape. The nonuniform bending stiffness is essential for the undulatory swimmer to generate nonreciprocal motion and thus provide propulsion [35–38]; the streamlined body reduces its resistance in the fluidic environment. The propulsion is generated by the tailbeat during the undulatory motion through fluid–robot interaction. With optimized structure design and actuation strategy, the resulting miniature soft swimmer performs efficient propulsion in the water and achieves a swimming velocity of 30 cm/s. Furthermore, the swimming ability of the swimmer in the 3-dimensional space is demonstrated by the vertical propulsion in the water. To demonstrate the feasibility of the swimmer for potential in vivo application, the efficient upstream swimming of the swimmer in a tubular environment is validated, and the ultrasound-guided navigation of the swimmer is implemented in a blood vessel phantom.

## Materials and Methods

### Design of the swimmer

**Fig. 1.**
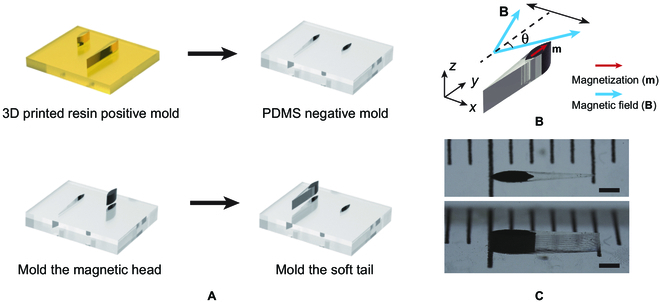
Design and fabrication of the miniature swimmer. (A) Schematics of the fabrication process. (B) Schematics of the actuation strategy. The blue arrow indicates the direction of the oscillating magnetic field, and the red arrow indicates the magnetization direction. (C) The top view and side view of the miniature swimmer. Scale bar: 1 mm.

The fish-like soft swimmer is designed to have a streamlined body. The swimmer is composed of a magnetic head and a nonmagnetic tail. The magnetic head is casted with a mixture of polydimethylsiloxane (PDMS) and neodymium–iron–boron (NdFeB) microparticles that have an average diameter of 5 μm. The soft tail is casted with pristine PDMS. For better clarification, we define the 3 axes of the swimmer as lateral axis, longitudinal axis, and vertical axis, corresponding to the *x*-axis, *y*-axis, and *z*-axis shown in Fig. 1B. Combining the variation in geometry and materials composition, the nonuniform bending stiffness along the longitudinal axis of the swimmer is obtained, which is important for generating thrust and improving energy efficiency. To fit in the biological environment of the human body, the swimmer is designed to have a maximum width of 0.5 mm, a height of 1 mm, and a length of 5 mm. The center of mass of the soft swimmer is arranged to be 32% of the total body length from its head.

### Fabrication of the swimmer

Since the swimmer is designed with an ultrathin feature (i.e. the end of the tail), a PDMS double-casting method is adopted to mold the swimmer (Fig. 1A). The positive mold is 3D-printed with a projection micro-stereolithography printer (BMF P150). The printed resin mold is heated in an oven at 80 °C for 2 h for complete curing. To cast the PDMS negative mold, the PDMS prepolymer (base:crosslinker = 10:1) is poured into the mold and cured in an oven at 60 °C for 2 h. After curing, the PDMS mold is gently released from the resin mold. Since the swimmer is also composed of PDMS, the surface of the PDMS negative mold needs to be specially treated before it can serve as a mold for the subsequent step. For the surface treatment, the PDMS negative mold is firstly treated by the plasma for 30 s to oxidize the functional groups on its surface and then immediately placed in ethanol to passivate the surface to suppress further chemical bonding [39].

Since the swimmer is composed of 2 parts with different materials composition, the mold casting of the swimmer is also divided into 2 steps. After obtaining the PDMS negative mold, the magnetic head is firstly cast. For casting the magnetic head, a mixture of PDMS and NdFeB microparticles (mass ratio = 1:1.8) is poured into the PDMS mold for the magnetic head (Fig. 1) and cured in an oven at 80 °C for 1 h. The cured magnetic heads are then placed in the adjacent mold of the whole body, and the PDMS prepolymer without NdFeB microparticles is added to fill the tail part of the mold. A subsequent curing process in an oven at 80 °C for 1 h leads to the complete curing of the tail part and the binding between the tail and the head of the swimmer. To optimize the swimming performance, swimmers with different tail stiffness are fabricated by adjusting the mass ratio between the base and the crosslinker of the PDMS prepolymer. With mass ratios of 5:1, 10:1, and 20:1, we fabricated 3 prototypes, swimmer A, swimmer B, and swimmer C, respectively. As the mass ratio between the base and the crosslinker increases, the tail stiffness of the swimmer decreases. The Young’s modulus of the tails of swimmer A, swimmer B, and swimmer C is 1.68, 0.86, and 0.16 MPa, respectively. After mold casting, the robotic swimmers are magnetized with a magnetizer to saturation; the magnetization direction is along their longitudinal axis.

### Actuation of the swimmer

The swimmer is actuated by an oscillating magnetic field (Fig. 1B). The magnetic field is oscillating around the longitudinal axis of the swimmer, with an oscillating angle of *θ*, and an oscillating frequency of *f*. The oscillating magnetic field can be expressed as:

$$B\left(t\right)=A\sin\theta \sin\left(2\pi \mathrm{ft}\right){\overset{⌢}{e}}_{x}+A\cos\theta {\overset{⌢}{e}}_{y}$$

where ${\overset{⌢}{e}}_{x}$ and ${\overset{⌢}{e}}_{y}$ represent the unit vector in the *x*-axis and *y*-axis, respectively. With proper rotation matrixes, pitch angles can be applied to the oscillating magnetic field, and the locomotion of the swimmer can thus be extended to 3-dimensional space. The uniform magnetic field in the 3-dimensional space is generated by a customized 3-axial Helmholtz coil.

## Results

### Characterization of the swimming performance

**Fig. 2.**
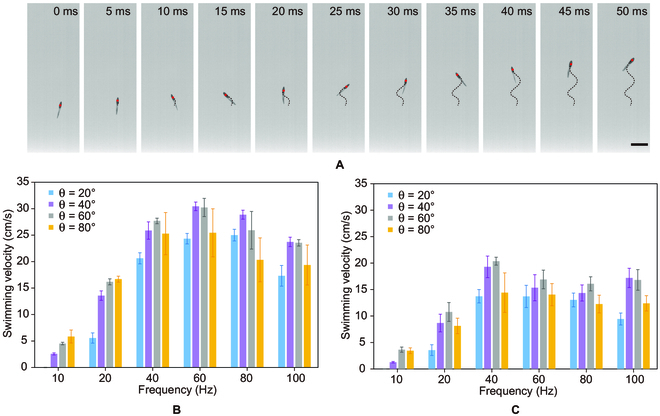
The swimming performance of the swimmer in the water. (A) Swimmer B swims in the water actuated by the oscillating magnetic field with a frequency of 40 Hz and an oscillating angle of 60°. The red point indicates the position of the swimmer, and the black dashed line indicates the trajectory of the swimmer. Scale bar: 5 mm. (B and C) The swimming velocity of swimmer B (B) and swimmer C (C) actuated by the oscillating magnetic field with frequencies *f* (*f* = 10 Hz, 20 Hz, 40 Hz, 60 Hz, 80 Hz, and 100 Hz) and oscillating angle (*θ* = 20°, 40°, 60°, and 80°).

To verify the swimming ability of the designed swimmer in the water, the swimming tests are implemented in a customized water tank inside the Helmholtz coil. Since the density of the swimmers is larger than water, the swimmers lie at the bottom of the tank in a steady state with its vertical axis horizontal to the bottom of the tank. Upon applying an oscillating magnetic field with a low frequency (<20 Hz), the robot starts oscillating in the magnetic field. Since the vertical axis of the swimmer is in the horizontal plane, the swimmer can only oscillate in its vertical plane, and no effective propulsion can be generated. When the frequency is larger than 20 Hz, self-righting behavior is observed, in which the swimmer can automatically reorient its vertical axis upright from the steady state and start swimming like a fish (Fig. 2A). In such configurations, the time-varying torque is applied to the magnetic head and generates a pitch motion. As the tail is soft and nonmagnetic, undulatory motion is thus generated arising from the nonuniform bending stiffness. The resulting vortices generated by the tailbeats provide propulsion for the swimmer. The self-righting behavior is triggered by the unevenly distributed flow field in the vertical direction when the swimmer is suddenly actuated and swings its tail. However, the self-righting behavior is only observed in swimmer B and swimmer C. Since the tail of swimmer A is softer than that of swimmer B and swimmer C, the fluid–structure interaction is not sufficient to reorient the body of the swimmer.

To characterize the swimming performance of the swimmer, swimmer B and swimmer C are adopted in the following tests. The swimming performance of these 2 swimmers is firstly evaluated in the static fluidic environment. Oscillating magnetic fields with different oscillation angles (*θ*) and oscillating frequencies (*f*) are applied to actuate the swimmers (Fig. 2 and Movie S1). As the swimmers are magnetized to saturation along their longitudinal axis, in an oscillating magnetic field, the head of the swimmers would oscillate to align with the magnetic field. By tuning the oscillating angle and frequency, the amplitude and frequency of the undulatory motion can be adjusted accordingly.

In the swimming tests, oscillating magnetic fields with frequencies ranging from 10 to 100 Hz and oscillating angles ranging from 20° to 80° are applied. The swimming motion of the swimmer is captured by a high-speed camera (Revealer 5F10) with a frame rate of 1,000 frames per second. The swimming velocities of swimmer B and swimmer C are plotted in Fig. 2B and C. As the oscillating frequency increases from 10 to 100 Hz, the swimming velocity first increases and then decreases, because high frequency leads to more tailbeat cycles but in the meantime also leads to step-out, which undermines the swimming performance. The oscillating angle also influences the swimming velocity. With a larger oscillating angle, it is easier for the swimmer to step out because of the larger stroke range in each cycle, while a smaller oscillating angle leads to a smaller propulsion force. For swimmer B, the maximum swimming velocity of 30 cm/s is achieved when the frequency is 60 Hz and the oscillating angle is 40°. Moreover, the swimming performance of swimmer B is overall better than that of swimmer C. The performance difference mainly comes from their difference in tail stiffness. As the tail of swimmer C is stiffer than that of swimmer B, the amplitude of the wave motion generated by swimmer C is smaller, which results in a smaller thrust. Besides, the stiffer body experiences more impedance in the undulatory motion, which results in a lower step-out frequency.

**Fig. 3.**
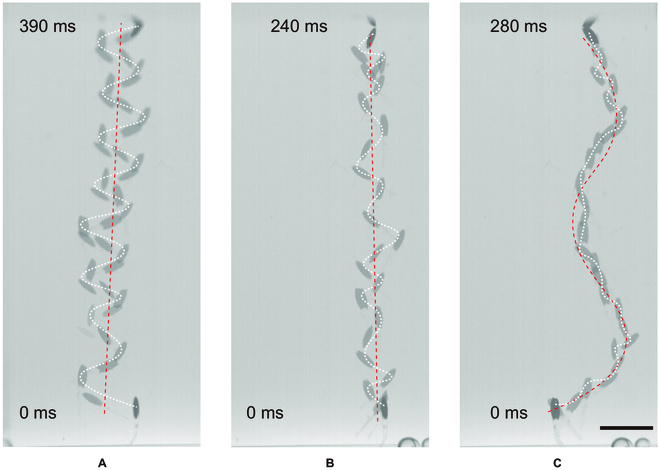
The swimmer swims vertically in the water. (A to C) The vertical swimming of the swimmer under the oscillating magnetic field with an oscillating angle of 20°, and oscillating frequencies of 20 Hz (A), 40 Hz (B), and 60 Hz (C). The red dashed line indicates the moving direction of the swimmer, and the white dashed lines indicate the trajectory of the swimmer. Scale bar: 5 mm.

The sufficient propulsion force generated by the swimmer also enables its vertical swimming in the water. In Fig. 2, we demonstrate the vertical swimming of swimmer B, under the oscillating magnetic field with an oscillating angle of 20° and oscillating frequencies of 20, 40, and 60 Hz, respectively (Fig. 3 and Movie S2). The maximum vertical swimming velocity in this scenario is achieved when the oscillating frequency is 40 Hz (Fig. 3B). The time-dependent configuration of the swimmer shows the trajectory of the swimmer under different oscillating frequencies. As the frequency increases, the amplitude of the undulatory motion decreases despite the oscillating angle of the magnetic field being the same, which indicates the step-out of the undulatory motion. As the frequency further increases to 60 Hz, the overall trajectory of the robot is not vertical but S-shaped because of more severe asynchronism.

Although self-righting behavior is not required for vertical swimming, there is still a threshold frequency. When the oscillating angle is set as 20°, an oscillating frequency larger than 20 Hz is required for effective vertical propulsion. Otherwise, the swimmer stays at the bottom of the tank due to insufficient propulsion force.

### Swimming in the confined space

**Fig. 4.**
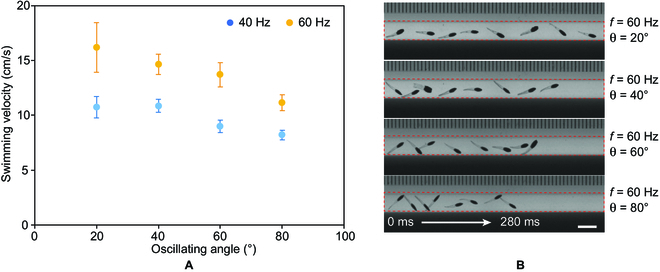
The swimming performance of swimmer B in a thin tube. (A) The swimming velocity of swimmer B in a thin tube. (B) The swimming process of the swimmer swimming across a thin tube under oscillating magnetic fields with different oscillating angles. The red dashed rectangle indicates the tube. Scale bar: 5 mm.

To evaluate the potential application of the swimmer in a confined space in the human body (e.g., in the blood vessel), swimming tests are conducted in a glass tube with a diameter of 4.1 mm. The frequencies of the oscillating magnetic fields are set as 40 and 60 Hz, and the oscillating angle of the magnetic field changes from 20° to 80°. As the amplitude of the undulatory motion is confined by the tube, the swimming performance is different from that in the open environment. Since the larger oscillating angle of the magnetic field leads to the larger amplitude of the undulatory motion, the swimming performance of the swimmer under an oscillating magnetic field with a larger oscillating angle is affected more by the geometry confinement. The highest swimming velocity is thus achieved when the oscillating angle of the magnetic field is set as 20°. As the oscillating angle increases, the swimming velocity decreases because of the collision and friction between the swimmer and the inner wall of the tube (Fig. 4B). The influence of the oscillating frequency on the swimming performance is not affected by the geometry confinement. Similar to the swimming tests in the open space, the higher oscillating frequency results in higher swimming velocity. Although the swimming of the swimmer in the confined space is undermined compared with that in an open space, the maximum swimming velocity can still reach 17 cm/s. Figure 4B shows the swimming process of the swimmer in the tube under the oscillating magnetic field with a frequency of 60 Hz and oscillating angles of 20°, 40°, 60°, and 80°, respectively.

### Upstream swimming

**Fig. 5.**
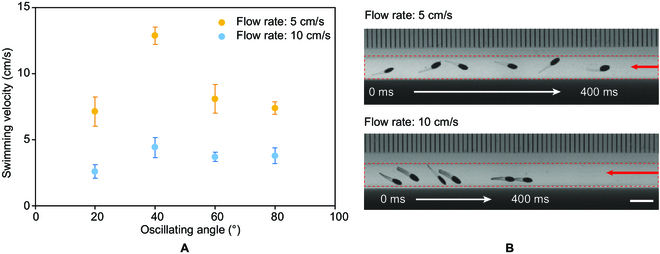
The upstream swimming performance of swimmer B in a thin tube. (A) The swimming velocity of swimmer B in fluid flow with flow speeds of 5 and 10 cm/s. (B) The swimming process of the swimmer swimming across a thin tube against the fluid flow with flow speeds of 5 and 10 cm/s, respectively. The red dashed rectangle indicates the tube. The red arrow indicates the direction of the flow. Scale bar: 5 mm.

Next, we test the ability of the swimmer to swim against the flow in a tubular environment. To generate a tubular environment with a flow, a glass tube with an inner diameter of 4.1 mm is connected to a peristaltic pump (Longer BT100-2J). The oscillating angle of the magnetic field is set as 20°, 40°, 60°, and 80°. The frequency of the oscillating magnetic field is set as 60 Hz. The swimming velocities of the swimmer in the flow with different flow speeds are measured as shown in Fig. 5A and Movie S3. Although the swimming test of the swimmer in the tube indicates that the swimming velocity of the swimmer decreases with the increasing oscillating angle, when the flow is introduced, the influence of the oscillating angle to the swimming performance is changed by the different fluid–structure interaction. The maximum velocity of the swimmer is achieved when the oscillating angle is 40°, because the coming flow suppresses the undulatory motion of the swimmer and reduces the collision and friction between the swimmer and the wall of the tube. When the flow speed is 5 cm/s, the maximum swimming velocity that can be achieved by the swimmer is 13 cm/s. As the flow speed increases to 10 cm/s, a swimming velocity of 4 cm/s can still be achieved. The ability of upstream swimming makes the swimmer a promising medical robot deployable in the blood vessel. When the flow velocity further increases from 10 to 15 cm/s, the velocity of the swimmer gradually decreases. As the flow velocity reaches 15 cm/s, we observed that the swimmer is not able to perform effective locomotion. It can only move around the starting position. Therefore, the maximum flow velocity that the swimmer can resist is about 15 cm/s. In the human body, the blood flow velocity in some regions exceeds 15 cm/s, and to effectively actuate the swimmer in such regions, the flow velocity of blood can be reduced using a surgical clamp, which is a common practice in surgery [40].

### Swimming in the biofluid

**Fig. 6.**
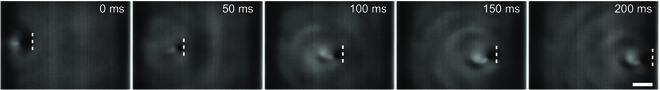
The swimming performance of the swimmer in the biofluid. The miniature swimmer swims in porcine whole blood. The white dashed line indicates the position of the swimmer. Scale bar: 5 mm.

In order to verify the swimming ability of the swimmer in the biofluid, we obtained porcine whole blood and conducted a swimming test in the blood (Fig. 6). Since the blood is not transparent, we can only observe the disturbance of the fluid. The swimmer is actuated by an oscillating magnetic field with a frequency of 60 Hz, an oscillating angle of 40°, and a magnitude of 20 mT. In the porcine whole blood, the swimmer can move with an average velocity of 11.6 cm/s. In general, the swimmer is capable of swimming in viscous blood, but the swimming velocity of the swimmer is smaller than that in the water. The decrease in swimming performance is mainly caused by the high viscosity of the blood.

### Ultrasound-guided navigation

**Fig. 7.**
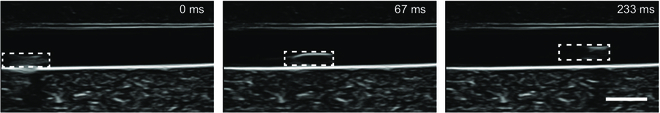
Ultrasound-guided locomotion of the swimmer. The miniature swimmer swims through a blood vessel phantom. The white dashed line indicates the position of the swimmer. Scale bar: 5 mm.

Since the imaging and navigation of miniature robots in the human body is a key challenge in the clinical application of untethered medical robots, we demonstrate the imaging and monitoring of the robot in a blood vessel phantom using an ultrasound device (Fig. 7 and Movie S4). The swimmer is placed inside the phantom and is actuated with an oscillating magnetic field with a frequency of 30 Hz and an oscillating angle of 30°. The swimmer can thus swim through the blood vessel phantom under ultrasound imaging.

## Discussion

In this work, we present a miniature soft swimmer with nonuniform bending stiffness and a streamlined body. The unique structure design endows the swimmer with large thrust and high energy efficiency. The swimming velocity of 30 cm/s is achieved, which is equivalent to 60 body lengths. With efficient undulatory swimming, the swimmer is also capable of swimming upward in the water. The swimming performance of the swimmer in the tubular environment is also investigated. The maximum swimming velocity of 17 cm/s is achieved in the tube with a diameter smaller than the body length of the swimmer. Toward the potential biomedical application, the swimming capability of the swimmer in fluidic flow is also validated. In the flow with a speed of 10 cm/s, a maximum swimming velocity of 5 cm/s can be maintained by the swimmer. Moreover, we demonstrate the ultrasound-guided navigation of the swimmer in a blood vessel phantom.

With the ultrafast swimming velocity and the ability of upstream swimming, the miniature soft swimmer we presented in this work shows great potential in targeted drug delivery, especially via the vascular system. Integrated with other imaging methods (e.g., digital subtraction angiography) and more dexterous electromagnetic devices, the robot can be implemented in the vascular system and rapidly navigated to the targeted region in the human body. The swimmer itself can serve as the carrier for drugs, and other responsive materials can also be integrated to release the drug on demand.

Toward the biomedical application, the biocompatibility and the upstream motility of the robot need to be further improved. For better biocompatibility, a biocompatible sealing layer can be grafted on the surface of the swimmer to reduce its toxicity, and an anticoagulation coating can be used to avoid the formation of thrombosis when implementing the swimmer in the blood vessel. To increase its controllability in regions with high blood flow velocity (e.g., >10 cm/s), we can either reduce the flow velocity by using the surgical clamp or introduce magnetic force generated by a gradient magnetic field. In conclusion, this work presents a prototype of a medical robot deployable in the vascular system. We will continue to improve the swimming performance and explore more functionalities of the robotic swimmer.

## Data Availability

All data are available in the main text or the Supplementary Materials.
